# Knowledge, attitudes, practices towards medication errors and adverse drug reactions reporting among healthcare professionals in Dongola, Sudan: a cross-sectional study

**DOI:** 10.1038/s41598-025-27221-4

**Published:** 2025-11-23

**Authors:** Mohammed A. Issak, Zeinab F. A. Mohamed, Kamal A. A. Mohammed

**Affiliations:** 1 Department of Pharmacology, Faculty of Medicine & Health Science, University of Dongola, Dongola, Northern State Sudan; 2 Department of Pharmacology, Faculty of Medicine & Health Science, University of Dongola, Dongola, PO Box47, 4111 Northern State Sudan

**Keywords:** Adverse drug reactions, Attitude, Dongola, Healthcare providers, Knowledge, Medication errors, Practice, Sudan, Health care, Medical research, Signs and symptoms

## Abstract

Medications, while beneficial, can also cause significant harm, often due to medication errors (MEs) and adverse drug reactions (ADRs). This study aimed to evaluate the knowledge, attitudes, and practices (KAP) of healthcare providers (HCPs) regarding ME and ADR reporting. A hospital-based cross-sectional study was conducted in Dongola and data were collected using a mixed-method approach with a semi-structured questionnaire. Analysis was performed using SPSS version 26, considering significance at *p* < 0.05. Out of 272 targeted HCPs, 216 responded (response rate: 79.4%). Most participants were female (71.8%) and aged ≤ 28 years (81%). For MEs reporting, average knowledge (59.3%), low attitudes (29.2%), and low practices (25%) were observed. Regarding ADRs reporting, knowledge was poor (35.2%), attitudes were positive (81.9%), and practices remained low (29.6%). Knowledge significantly associated with reporting practices for both MEs and ADRs, using Chi-Square Test (*p* < 0.001). Nurses showed better MEs reporting practices compared to physicians and pharmacists (*p* = 0.008). Positive attitudes significantly correlated with improved ADRs practices (*p* = 0.001). The primary barrier to reporting was lack of knowledge (28.9%), while the most suggested improvement was implementing a mandatory reporting system (37.5%). In conclusion, the participants had inadequate knowledge and practices regarding MEs and ADRs reporting, emphasizing the need for targeted interventions to improve reporting practices.

## Introduction

Medication errors (MEs) and adverse drug reactions (ADRs) represent critical challenges to patient safety, contributing significantly to global morbidity, mortality, and healthcare costs^1^. MEs are preventable events that can occur at any stage of the medication use process, including prescribing, transcribing, dispensing, administering, and monitoring^2^. Adverse drug reactions (ADRs), on the other hand is defined by the World Health Organization (WHO) as “any noxious or unintended effect produced by a drug at standard therapeutic doses,”^3^. The process of medication in general commences with a doctor’s prescription, followed by the pharmacist’s check-up and the medication is finally administered to the patient by a nurse or a medical staff. ME and ADRs can occur at any point in the medication use process, including prescribing, transcribing, dispensing, administering, and monitoring medications^4^.

MEs and ADRs are often interrelated, as approximately 25% of ADRs are caused by MEs and are therefore the leading preventable cause of it^5^.Together, these issues place a substantial burden on healthcare systems. with costs exceeding $40 billion annually, representing nearly 1% of all healthcare expenditures worldwide^6^. Beyond financial implications, they erode patient trust, prolong hospital stays, and increase healthcare utilization^6^.

Despite their impact, underreporting of MEs and ADRs by healthcare professionals (HCPs) remains a significant barrier to improving medication safety^7^. Globally, only 2% of ADRs are reported, with barriers such as lack of time, knowledge, feedback, and fear of repercussions^8,9^. In response, interventions such as education, training, and the implementation of user-friendly electronic reporting systems have proven effective in enhancing reporting rates^10^. Recognizing the global burden of medication-related harm, the WHO initiated the Global Patient Safety Challenge in 2017 to reduce medication-related harm by 50% by 2022^11^. However, progress in Africa remains limited, with only 37 countries participating in the WHO Programme for International Drug Monitoring (WHO PIDM) and a minimal contribution of ME reports to global databases^12^. In Sudan, the situation is particularly concerning. Despite being a member of the WHO PIDM since 2008, the country has a zero reporting rate for MEs and weak ADR reporting practices, primarily due to the absence of legal obligations and systemic barriers^13^.

Many studies revealed significant variations in HCPs’ knowledge, attitudes, and practices (KAP) toward MEs and ADRs reporting, and the importance of fostering a culture of reporting, and dealing with the identified barriers such as lack of feedback, and training^14^. Although most of healthcare professionals demonstrate positive attitudes toward reporting, actual reporting practices remain low due to systemic and individual challenges. For instance, a study from Saudi Arabia revealed that 90% of participants had favorable attitudes, but 44.8% had never reported MEs^15^. Another study from Pakistan reported average knowledge (45.7%), favorable attitude (89.9%), and poor practices (28.2%)^16^. The situation is similarly concerning regarding ADR reporting, with several studies from Ethiopia, India, and Pakistan reporting poor knowledge and low reporting practices despite generally positive attitudes among healthcare professionals^17,19^.

Barriers to reporting MEs and ADRs include fear of consequences, heavy workloads, and lack of knowledge about how and where to report^9^. On the other hand, strategies suggested to promote reporting include education, provision of accessible systems, and fostering a blame free and non-punitive environment^10^.

To date, there is limited data about the KAP of HCPs regarding ME and ADR reporting, the barriers hindering the reporting process, and the strategies for improving it. Investigating and understanding these issues is critical to identifying gaps, informing the relevant authorities for proper and timely interventions, ultimately improving patient safety. Therefore, this study aims to assess the KAP of HCPs in Dongola, Sudan, toward ME and ADR reporting, identify common barriers, and propose strategies to enhance reporting practices.

## Methods

### Study design and setting

The study used descriptive and analytical cross-sectional hospital-based design, conducted at the main hospitals in Dongola, the capital city of the Northern state of Sudan, in the period from December 2023 to February 2024.The study strictly adhered to the cross-sectional reporting guidelines of “Strengthening of Reporting of Observational Studies in Epidemiology” (STROBE) to ensure methodological rigor^20^.

### Study population

The study targeted the healthcare professionals (physicians, pharmacists and nurses) working in main hospitals in Dongola, Northern State, Sudan, with a total of 680 staff. The study included any healthcare professional officially working in one of the main hospitals in Dongola city during the study period, who provided consent for participation in the study.

Sample size was calculated using the Yamane’s formula,$\:Sample\:size\:\left(n\right)=\frac{\varvec{N}}{1+(\varvec{N}\times\:{\varvec{e}}^{2})}$, considering a total population (N) of 688, confidence level of 95%, a marginal error (e) of 5%, and a proportion of 50%. The calculated sample size was 247 and was increased by 10% to ensure a greater chance of generalization and compensate for low and missing responses. The final sample size was 272 with a sampling technique of convenience sampling procedure.

### Study variables

The independent study variables were the sex, age, profession, and experience years. The dependent variables were the knowledge, attitude, and practice of the participants towards the reporting of MEs, and ADRs.

### Data collection instruments

Two semi-structured questionnaires adopted from several previously published studies were used in the current study.15–18 The two questionnaires consisted of 35 items in total, each grouped into FIVE sections: Socio-demographical Sect. (5 items); knowledge Sect. (5 items); attitude Sect. (5 items), practice Sect. (5 items) and common barriers of reporting and ideas for improving Sect. (2 items).

The questionnaires were translated into Arabic to enhance comprehension, as it is the primary language of the participants. For content validity, an expert in the researched area (PhD in Pharmacy) reviewed the questionnaire. The questionnaire was modified according to the expert’s comments. In addition, the data was statistically assessed for reliability and both showed Cronbach’s alpha values of 0.78 for KAP towards Medication Error Reporting, and 0.65 for KAP towards Adverse Drug Reaction reporting. As reported in previous studies. a Cronbach’s alpha value of above 0.6 is acceptable and considered reliable^21^.

### Data collection

Data were collected via printed forms, interviews, and Google Forms during hospital break times. The study objectives were clearly stated in the questionnaire. To maintain consistency, all data collection was conducted personally by the researchers.

### Pilot testing

A pilot test was conducted with 15 healthcare professionals (7 physicians, 3 pharmacists, 5 nurses) from the different targeted hospitals to assess the overall understandability and clarity of the instrument for the targeted participants using both questionnaires.

### Data management

The data was analyzed using the Statistical Package for Social Science (SPSS), version 26. For each domain (knowledge, attitude, and practice), scores were calculated by summing the correct or positive responses. To categorize the results, a cutoff point of 50% of the maximum possible score was used to distinguish between “good” (≥ 50%) and “poor” (< 50%) levels. This threshold was selected based on its frequent use in similar KAP studies among healthcare providers, where it is considered to represent a basic competency level in the measured domain.

Descriptive and comparative analyses were performed, and are presented in form frequencies and percentage. Non-parametric Mann-Whitney and Kruskal-Wallis Test was used to assess the significance associations and their magnitude, considering a p value level of ≤ 0.05, since the data was not normally distributed.

To ensure data collection accuracy and keeping the privacy rights of the participating individuals, the personal identification information collected was neither used nor presented in this study and confidentiality of the data was maintained by using serial number.

### Ethical consideration

The study was conducted in compliance with the Declaration of Helsinki. Ethical approval was obtained from the Research Ethical Committee of the Northern State Ministry of Health (REC-MoH-NS-8-2023) and the research offices of the participating hospitals. Written informed consent was obtained from all participants prior to completing the questionnaire. Ethical clearance certificates and a signed sample of the consent forms are available for review upon request.

## Results

### General characteristics of the study population

The study included 216 healthcare professionals, predominantly female (71.8%), with a median age of 26 years (IQR: 25–28). Physicians constituted more than two-third (69.4%), and had experience of less than 3 years (69.4%). These data are summarized in Table 1.

**Table 1:** Socio-Demographics of the study Participant, 2023.

Characteristics	Frequency	Percent
Sex		
Male	61	28.2
Female	155	71.8
Age (years)^*^		
≤ 28 years	175	81.0
> 28 years	41	19.0
Profession		
Doctor	127	58.8
Nurse	77	35.6
Pharmacist	12	5.6
Experience years		
< 3 years	150	69.4
3–5 years	41	19.0
> 5 years	25	11.6

*Median (IQR) Age: 26 (IQR of 25–28).

### Knowledge, attitude, and practices of the study population towards MEs reporting

Good knowledge of ME reporting was observed in 59.3% of participants. Only about half correctly identified the components of medication errors (52.8%), and accepted the responsibilities of reporting it (53.2%).While 67.1% recognized the importance of educational interventions in reducing errors, only 34.3% believed intercepted errors should be reported, and 38.9% understood the financial burden MEs impose on healthcare systems. Details of participants’ knowledge are summarized in Table 2.

**Table 2:** Knowledge of the study participant towards ME, 2023.

Statement	Frequency	Percent (%)
Options	Yes	No
1. Do medication errors include wrong dosage, wrong route, and wrong documentation?	114 (52.8%)	102 (47.2%)
2. Can educational interventions and training courses reduce medication errors?	145 (67.1%)	71 (32.9%)
3. Do all HCPs have responsibilities in preventing the occurrence of medication errors?	115 (53.2%)	101 (46.8%)
4. Should a healthcare professional report an error where medication was prevented from reaching the patient?	74 (34.3%)	142 (65.7%)
5. Do medication errors play critical role in increasing healthcare system costs?	84 (38.9%)	132 (61.1%)
Overall ME knowledge score		
Good		128 (59.3%)
Poor		88 (40.7%)

Attitudes toward ME reporting were predominantly negative, with 70.8% scoring poorly. Despite 83.3% agreeing that reporting enhances patient safety, 66.7% believed intercepted errors did not require reporting, and 81.9% denied their reporting responsibilities. About half reported worries about lack of confidentially in the reporting process (49.1%), and some expressed fear of blame when reporting their own errors (42.1%). These findings are presented in Table 3.

**Table 3:** Attitude of the study participant towards ME, 2023.

Statement	Frequency	Percent (%)
Options	Agree	Disagree
A. Medication errors do not need to be reported if detected before reaching the Patient	144 (66.7%)	72 (33.3%)
B. Information I disclose when reporting a medication error will be Confidential	106 (49.1%)	110 (50.9%)
C. It is not my responsibility to report medication errors caused by someone else	39 (18.1%)	177 (81.9%)
D. I fear being blamed if I report a medication error I made	91 (42.1%)	125 (57.9%)
E. Reporting medication errors contributes to the continuous improvement in patients’ safety	180 (83.3%)	36 (16.7%)
Overall ME attitude score		
Positive	63 (29.2%)	
Negative	153 (70.8%)	

Good ME reporting practices were reported by only 25.0% of participants. Most (84.7%) had engaged in training or self-study on ME management. However, only 38.4% consistently reported intercepted errors, and 47.2% reported errors made by colleagues. These data are detailed in Table 4.

**Table 4:** Practice of the study participant towards ME, 2023.

Statement	Frequency	Percent (%)
Options	Yes	No
1. I report as a medication error if the patient is prescribed an inadequate dose	117 (54.2%)	99 (45.8%)
2. I report as a medication error even if it is detected before reaching the Patient	83 (38.4%)	133 (61.6%)
3. I attend training courses or read about medication errors and their management	183 (84.7%)	33 (15.3%)
4. I consult with the senior physicians in medication error cases that I am not sure	124 (57.4%)	92 (42.6%)
5. I report the medication errors that my colleagues make	102 (47.2%)	114 (52.8%)
Overall ME practice score		
Good		54 (25.0%)
Bad		162 (75.0%)

### Knowledge, attitude, and practices of the study population towards ADRs reporting

Knowledge of ADRs reporting was generally poor, with 64.8% scoring poorly. Although 81.9% knew the National Medicines and Poisonous Board was the reporting authority, only few correctly defined ADRs (23.6%), and identified its correct components (14.4%). Only about half acknowledged the difference between side effects and ADR (47.2), and identified renal failure as a major risk factor for ADRs (50.5%). Comprehensive findings are shown in Table 5.

**Table 5:** Knowledge of the study participant towards ADR, 2023.

Question	*n* (%)
1. Which of the following defines ADR correctly	
1. Any noxious or undesired effect of drug occurring at normal dose, during normal use	51 (23.6%)
2. Harm resulting from the use of substandard or counterfeit drugs	94 (43.5%)
3. Harm caused by drug overdose	71 (32.9%)
2. Which ADR should be reported?	
1. Any noxious or undesired effect of drug occurring at normal dose, during normal use	32 (14.8%)
2. ADRs to vaccines only	33 (15.3%)
3. Unknown ADRs to old and new drugs only	120 (55.6%)
4. All of the above	31 (14.4%)
3. Where are ADRs reported in Sudan?	
1. National Medicines and Poisonous Board	177 (81.9%)
2. Federal Ministry of Health	39 (18.1%)
4. Is ADR the same as side effects?	
1. Yes	114 (52.8%)
2. No	102 (47.2%)
5. The following is the major risk factor for the occurrence of maximum ADRs?	
A. Renal failure	109 (50.5%)
B. Visual impairment	2 (0.9%)
C. Both	105 (48.6%)
Overall ADR Knowledge Score	
Good	76 (35.2%)
Poor	140 (64.8%)

Attitudes toward ADR reporting were mostly positive, with 81.9% demonstrating favourable attitude. Majority agreed that ADR reporting is necessary (81.5%) and enhances patient safety (88.4%). Similarly, most of the respondents agreed to the importance of establishing reporting centers in all hospitals (78.2%), and making the reporting practice mandatory for all HCPs (75.0%). However, about half expressed the process of reporting as time-consuming (48.1%). Detailed results are summarized in Table 6.

**Table 6:** Attitude of the study participant towards ADR, 2023.

Statement	*n* (%)	*n* (%)
Options	Agree	Disagree
A. ADR reporting is necessary and important for healthcare system	176 (81.5%)	40 (18.5%)
B. ADR reporting should be mandatory for all HCPs	162 (75%)	54 (25%)
C. ADR reporting increase patient’s safety	191 (88.4%)	25 (11.6%)
D. ADR reporting creates additional workload and it is time consuming	104 (48.1%)	112 (51.9%)
E. Establishing ADR reporting center in every hospital is important	169 (78.2%)	47 (21.8%)
Overall ADR Attitude Score		
Positive	177 (81.9%)	
Negative	63 (29.2%)	

Good ADR reporting practices were demonstrated by only 29.6% of participants. While 81.5% counseled patients on ADRs, just 14.4% consistently reported them. About two-third expressed hesitance regarding ADR reporting (62.5%), and about half did not consult with their seniors in ambiguous ADR cases (49.5%). Only a few reported attending ADR courses and reading ADR related articles (23.6%).The practice-related findings are presented in Table 7.

**Table 7:** Practice of the study participant towards ADR, 2023.

Statement	Frequency	Percent (%)
Options	Yes	No
A. I attend courses and read articles in relation to prevention of ADR	51 (23.6%)	165 (76.4%)
B. I provide appropriate counseling for the patients regarding the possible ADRs associated with drugs	176 (81.5%)	40 (18.5%)
C. I report any ADR immediately and directly to the responsible authority	31 (14.4%)	185 (85.6%)
D. I sometimes hesitate to report ADR due to concerns about time it takes to report	135 (62.5%)	81 (37.5%)
E. I consult with the senior physicians in ADR cases that I am not sure	109 (50.5%)	107 (49.5%)
Overall ADR practice score		
Good		64 (29.6%)
Bad		152 (70.4%)

### Factors influencing the practice of the study population regarding ME and ADR reporting

Knowledge was significantly correlated with reporting practices for both MEs and ADRs (*p* < 0.001). Nurses demonstrated better ME reporting practices compared to physicians and pharmacists (*p* = 0.008). Positive attitudes was significantly correlated with better ADR practices (*p* = 0.001). These results are summarized in Table 8.

**Table 8:** Correlation of basic characteristics of the studied participants with ME and ADR reporting Practices, using non-parametric Mann-Whitney and Kruskal-Walis Tests, 2023.

Variable	Practice of ME reporting		Practice of ADR reporting	
	Mean rank	*P*-value	Mean rank	*P*-value
Sex				
Male	106.60	0.734^*^	113.02	0.471^*^
Female	109.39		106.72	
Age				
≤ 28 years	108.73	0.911^*^	105.67	0.136^*^
> 28 years	107.54		120.60	
Profession				
Doctor	97.76	0.008^**+**^	103.89	0.275^**+**^
Nurse	125.11		102.38	
Pharmacist	115.58		117.06	
Years of experience				
< 3 years	110.82	0.617^**+**^	105.07	0.376^**+**^
3–5 years	100.24		113.52	
> 5 years	108.14		120.86	
Knowledge about ME and ADR				
Good	141.88	< 0.001^*^	171.24	< 0.001^*^
Poor	59.94		74.44	
Attitude towards ME and ADR				
Positive	113.35	0.454^*^	135.13	0.001^*^
Negative	106.50		102.63	

*: Mann-Whitney Test **| +**: Kruskal Wallis Test.

### Barriers to and suggestions for improving the ME and ADR reporting practices among the studied population

Barriers to reporting included lack of knowledge (28.9%) and absence of feedback or reporting centers (26.2%). Participants suggested making reporting systems mandatory (37.5%) and establishing accessible reporting system and centers (27.1%). Detailed findings are summarized in Table 9.

**Table 9:** Reasons for under-reporting and suggestions for improving the reporting practices from the study participant, 2023.

Variable	Frequency	Percent (%)
Reasons for under-reporting^*^		
1. Reporting is unnecessary and time wasting	20	7.8
2. The reporting system is not mandatory in the state	59	23.0
3. The reporting forms are too complicated	16	6.2
4. Lack of knowledge about it	74	28.9
5. Lack of time for reporting	20	7.8
6. Lack of reporting center or feedback	67	26.2
Ideas for improving the reporting practices^*^		
1. Regular educational training about it	24	8.3
2. Making the reporting system mandatory among the HCPs	107	37.5
3. Making reporting system easy and accessible	77	27.1
4. Providing reporting centers and forms in every hospital	77	27.1

*Multiple Response Analysis (a respondent could choose one or more options or none at all).

## Discussion

While medications offer numerous benefits, they can also pose significant risks topeople^2^. MEs and AD Rs are critical factors contributing to this harm.2 This study aims to provide a comprehensive overview of healthcare providers’ knowledge, attitudes, and practices regarding the reporting of MEs and ADRs.

A total of 246 HCPs from government hospitals in Dongola were invited to participate in the study, with 216 voluntarily participating, resulting in a response rate of 79.4%, which is considered acceptable for global online surveys^22^.

Understanding MEs is essential for effective reporting. The current study found an average knowledge rate of 59.3% among participants. Comparatively, studies from Palestine, Pakistan and Malaysia reported almost similar average knowledge rates of 51.5%, 45.7% and 41.2%, respectively^16,23,24^. In contrast, a study from Saudi Arabia indicated a significantly higher knowledge score of 97%.^15^ This discrepancy may be attributed to the fact that the Saudi study focused solely on physicians, while our study included nurses and pharmacists, many of whom may have less experience.

Regarding MEs attitudes, it was shown to be very low (29.2%). On the contrary, the study from Saudi Arabia reported a much higher favorable attitude of 90%^15^. The lower attitudes rates of the HCPs in the current study could be attributed to their insufficient knowledge. The study revealed a low favorable attitude rate of 29.2% towards MEs. Conversely, the Saudi study reported a much higher favorable attitude of 90%^15^. The lower attitude rates in our study may be linked to insufficient knowledge among healthcare providers.

The practice rate for reporting MEs in our study was 25%, which aligns closely with the28.2% reported in Pakistan^16^. The low reporting practices observed in our study and similar studies in developing countries may stem from weaknesses in the healthcare system.

The knowledge score regarding ADRs in our study was 35.2%, which is lower than the58.3% reported in a previous study from Ethiopia^17^. This difference may be due to the fact that nearly half of Ethiopian participants had received training on AD R reporting, while the majority of participants of the current study denied attending courses related to ADR prevention.

The attitude towards ADRs reporting in our study was 81.9%, which is consistent with previous studies from Ethiopia and Pakistan, reporting 73.6% and 78.2%,respectively^17,18^. However, the practice rate for ADRs reporting in our study was 29.6%,slightly lower than the 32.1% reported in Ethiopia, indicating a regional challenge^17^.

Our findings indicate a significant correlation between knowledge and reporting practices for both MEs and ADRs (*p* < 0.001). This is also reported in previous studies^17,25^. Nurses in the current study demonstrated better reporting practices for MEs compared tophysicians and pharmacists (*p* = 0.008). Positive attitudes were also significantly correlatedwith better ADR reporting practices (*p* = 0.001), although these were not observed in theprevious literature.

Under- reporting of MEs and ADRs in our study was primarily attributed to a lack of knowledge (28.9%). Similar findings were reported in studies from Ghana andEthiopia^26,27^. Other barriers included the absence of reporting centers and feedback mechanisms (26.2%), as well as busy timetable (7.8%), which were also noted in previousstudies^23,27–29^

Participants suggested several strategies to enhance reporting practices. A significant(37.5%) of participants advocated for making reporting mandatory among healthcare providers. While this remains a topic of debate, it underscores the need for integrating reporting into routine healthcare practices^10^. Participants also suggested that reporting systems should be made more accessible and user- friendly (27.1%). Previous studies have stressed that an efficient error reporting system is essential for improving the reporting practices^30–32^. Regular education and training for healthcare personnel on identifying and reporting errors were also emphasized. A recent Cochrane review supported this approach, demonstrating the importance of educating the HCPs’ about the MEs and ADRs and the benefits of reporting them^32^.

This study has several limitations. Although the findings are specific to government hospitals in Dongola, the healthcare providers working in these hospitals come from diverse communities, offering the possibility of some generalizability to other settings. The cross-sectional design captures a single point in time, limiting insights into changes in knowledge, attitudes, and practices over time. Additionally, while self-reported data can introduce bias, this was minimized by using a mixed-method approach to data collection, which included an online Google form, and printed questionnaires.

Recommendations.

To enhance medication error (ME) and adverse drug reaction (ADR) reporting among healthcare professionals, the following actions are recommended:

1. Integrate pharmacovigilance and patient safety topics into undergraduate and postgraduate health curricula.
2. Conduct regular continuing professional development (CPD) sessions to improve awareness and reporting skills.
3. Establish simple and accessible electronic reporting systems with timely feedback mechanisms.
4. Implement mandatory but non-punitive reporting policies to foster a culture of learning rather than blame.
5. Strengthen collaboration between healthcare institutions and the National Medicines and Poisons Board to ensure effective data exchange and response.

## Conclusion

This study demonstrates critical gaps in the knowledge, attitudes, and practices of healthcare providers regarding MEs and ADR reporting in Dongola, Sudan. While knowledge and attitudes showed some variability, the reporting practices were alarmingly inadequate, raising significant concerns for patient safety. The identified barriers, including insufficient knowledge, lack of reporting systems, and busy timetables due to heavy workload, further complicate these issues. There is urgent need for interventions to enhance HCPs’ competencies and reporting practices.

### What is already know on this topic

- Medication errors (MEs) and adverse drug reactions (ADRs) are global healthcare challenges that significantly contribute to morbidity, mortality, and financial burdens.
- Despite positive attitudes among healthcare professionals, the actual reporting rates of MEs and ADRs remain low due to various systemic and individual barriers.
- Strategies like education, providing user-friendly reporting systems, and fostering a non-punitive reporting environment have proven effective in enhancing reporting rates.

### What this study adds

- This study reveals a low knowledge score (59.3% for MEs and 35.2% for ADRs), varying attitude levels (29.2% for MEs and 81.9% for ADRs) among healthcare providers in Dongola, Sudan.
- The reporting practices for both MEs (25%) and ADRs (29.6%) remain poor among healthcare providers in Dongola, Sudan.
- The primary reasons for underreporting were insufficient knowledge of how, what and where to report (28.9%), whereas the main proposed strategy for disproving the reporting practices was making reporting mandatory(37.5%).

## Data Availability

The datasets generated and/or analyzed during the current study are available from the corresponding author on reasonable request.
